# Association of patient comorbidities with colorectal cancer site as detected by computed tomography scan and colonoscopy: A retrospective study

**DOI:** 10.1097/MD.0000000000040711

**Published:** 2024-11-22

**Authors:** Jehad Fataftah, Naser El-Hammuri, Maha Gharaibeh, Mohammad Badran, Aqleh Ibrahim, Basil Alramahi, Abdel Rahman Alfawadleh, Ayman Alqubelat

**Affiliations:** a Radiology Department, Faculty of Medicine, The Hashemite University, Zarqa, Jordan; b Al Seef Hospital, Salmiya, Kuwait; c Radiology Department, Faculty of Medicine, Jordan University of Science and Technology, Irbid, Jordan; d Faculty of Medicine, The Hashemite University, Zarqa, Jordan.

**Keywords:** abdominal computed tomography, colonoscopy, colorectal cancer, comorbidities, diabetes mellitus, hypertension

## Abstract

Colorectal cancer (CRC) ranks as the third most common cancer worldwide. The most prevalent site is sigmoid. Comorbidities are common in patients with CRC and may be an important prognostic factor. This study investigated the prevalence of comorbidities among patients with CRC and assessed the association between the number of comorbidities and various factors including age, tumor site, smoking status, cancer stage, and mortality. This retrospective study included patients diagnosed with cancer at our hospital between January 2018 and November 2021. The association between comorbid illnesses (diabetes mellitus and hypertension) and patient characteristics such as sex, marital status, and smoking status was evaluated. The impacts of comorbid illnesses on CRC diagnosis and outcomes (cancer stage, primary site, and patient status) were analyzed. A chi-square test was performed to assess the relationship between sex, marital status, smoking status, and the presence of comorbid conditions (diabetes mellitus and/or hypertension). The majority of patients had at least 1 comorbid illness. A higher proportion of males had comorbid conditions compared to females. The proportion of patients with an early-stage cancer was higher among those without comorbidities. The proportion of surviving patients was higher among those without comorbidities, suggesting that patients with comorbid conditions may have an increased risk of death. The sigmoid colon was the most common site for colon cancer. Hypertension was the most common comorbidity followed by diabetes mellitus. Almost 50% of patients with CRC were smokers.

## 1. Introduction

The global incidence of new cases and mortality associated with colorectal cancer (CRC) continues to increase each year, making CRC one of the most frequently diagnosed cancers in both men and women in the United States of America.^[1]^ Comorbidities associated with chronic diseases are also a growing concern. It is projected that approximately 75% of all global deaths by 2030 will be attributed to noncommunicable diseases. Given the distinct anatomic and functional role of the large intestine and colon, recognizing how CRC affects these areas can provide deeper insights into its prevalence and the associated comorbidities.

The location of colorectal malignancies, based on colonoscopy and computed tomography findings, include rectal cancer (49.66%), colon cancer (49.09%), and both sites together (1.25%). Among colon cancers, the most prevalent sites are the sigmoid colon (55%), ascending colon (23.3%), transverse colon (8.5%), descending colon (8.1%), cecum (8.0%), and the crossing site (2.1%).^[2]^ Colorectal cancer ranks as the third most common cancer worldwide, and the second leading cause of cancer-related deaths.^[3]^ It is more prevalent in men than in women and is 3 to 4 times more common in developed countries than in developing countries. Colorectal cancer comprises a heterogeneous group of diseases caused by numerous mutations and mutagens.^[4]^ Colorectal cancer is caused by mutations in oncogenes, tumor suppressor genes, and genes involved in DNA repair mechanisms. Depending on the origin of the mutation, colorectal carcinomas can be categorized as sporadic (70%), hereditary (5%), or familial (25%).^[5]^ Colorectal cancer is predominantly diagnosed in older adults, with 60% of newly diagnosed cases in patients aged 70 years or older.

Comorbidities are common in patients with CRC and may be an important prognostic factor.^[6]^ These patients often face frailty and multiple comorbidities which necessitate comprehensive evaluation and stratification.^[7]^ Thus, understanding the impact of comorbidities and other factors on survival is crucial for effective cancer management, particularly in the aging population. As older patients with high comorbidities are often excluded from clinical trials, trial-based evidence for optimal therapy in these cohorts is limited, leading to increased reliance on observational studies.^[8]^ Hypertension, diabetes mellitus, and gastric diseases are common comorbidities associated with colon and rectal cancers and medications are often prescribed for hyperlipidemia, hypertension, and gastroesophageal reflux disease.^[9]^

This study investigated the prevalence of comorbidities among patients with CRC and assessed the association between the number of comorbidities and various factors including age, tumor site, smoking status, cancer stage, and mortality.

To address the existing gap in understanding the impact of comorbidities on CRC prognosis, in this study we assessed the prevalence of comorbidities among patients with CRC and the association between the number of comorbidities and various factors including age, site of tumor site, smoking status, cancer stage, and mortality.

## 2. Materials and methods

### 2.1. Study design

In this retrospective study, statistical data obtained from the Prince Hamzah Hospital system in Amman, Jordan were analyzed to investigate statistical patterns that could aid in distinguishing various risk groups among patients. Data included consultations, colonoscopy results, and histopathological and immunohistochemical analyses performed in the hospital laboratory, as well as patient demographics, medical records, and treatment plans.

### 2.2. Data collection

All patients underwent colonoscopy for suspected colorectal cancer at Prince Hamza Hospital between January 2018 and November 2021 and diagnosed with cancer were included in the study. Biopsies were obtained during each procedure and tumor samples were processed in the histopathology lab at the hospital. Data regarding patient demographics (age, sex, and origin) and medical information (medical records and treatment plans) were retrieved from the hospital system (Hakim) with approval from the Prince Hamza Hospital Research Committee.

### 2.3. Statistical analyses

Data were entered into a Microsoft Office Excel (Microsoft Corp., Redmond, WA) database and processed using IBM SPSS Statistics version 23.0 (IBM Corp., Armonk, NY). Descriptive statistics were used to summarize patient demographics and clinical characteristics and obtain a general understanding of their distributions and patterns. The associations between comorbid illnesses (diabetes mellitus [DM] and hypertension [HTN]) and patient characteristics such as (sex, marital status, and smoking status) were evaluated. The impacts of comorbid illnesses (DM and HTN) on CRC diagnosis and outcomes (cancer stage, primary site, and patient status) were analyzed.

Comparative analyses were performed to assess the relationships between various patient characteristics, comorbidities, and colorectal cancer outcomes. Chi-square tests evaluated the associations between categorical variables. The significance level was set at .05, and analyses were performed using a 95% confidence level.

Chi-square tests were used to evaluate the associations between sex, marital status, smoking status, cancer stage, primary cancer site, patient status and the presence of comorbid conditions (DM and HTN). Although this study identified sex as a significant factor associated with the presence of comorbid conditions, no specific adjustments for potential confounders were made in the analyses.

## 3. Results

### 3.1. Descriptive statistics

Table 1 presents a comprehensive overview of the demographics, diagnoses, outcomes, and comorbidity characteristics of 100 patients diagnosed with colorectal cancer between January 2018 and November 2021. Of these patients, 34% were females and 66% were males. Most patients (78%) were married, and 45% were smokers.

**Table 1:** Characteristics and outcomes of patients with colorectal cancer.

No	Characteristic		Count	Percentage (%)
Demographic characteristics				
1	Sex	Males	66.0	66.0
		Females	34.0	34.0
2	Marital status	Married	78.0	78.0
		Not married	22.0	22.0
3	Smoking status	Nonsmoker	55	55.0
		Smoker	45	45.0
Diagnosis				
4	Cancer stage at diagnosis	1	36	36.0
		2	23	23.0
		3	25	25.0
		4	16	16.0
5	Primary site	Sigmoid colon	27	27.0
		Rectum	24	24.0
		Ascending colon	16	16.0
		Descending colon	13	13.0
		Rectosigmoid	11	11.0
		Transverse colon	5	5.0
		Splenic flexure	3	3.0
		Hepatic flexure	1	1.0
6	Type of cancer	Adenocarcinoma	93	93.0
		Burkitt lymphoma	2	2.0
		Carcinoid	2	2.0
		Neuroendocrine carcinoma	2	2.0
		Squamous cell carcinoma	1	1.0
7	Age at time of diagnosis (years)	˂60	39	39.0
		60–70	33	33.0
		>70	28	28.0
Outcomes				
8	Length of stay at hospital (days)	˂10	22	22.0
		10–14	39	39.0
		>15	4	4.0
		Outpatient clinics	35	35.0
9	Status	Alive	78	78.0
		Dead	22	22.0
Comorbidity				
10	Presence of DM	Yes	40	40.0
		No	60	60.0
11	Presence of HTN	Yes	53	53.0
		No	47	47.0
12	DM diagnosis age (years)	˂60	17	42.5
		60–70	18	45.0
		>70	5	12.5
13	HTN diagnosis age (years)	<60	20	37.7
		60–70	24	45.3
		>70	9	17.0

DM = diabetes mellitus, HTN = hypertension.

Regarding diagnosis, the majority of cancers (93%) were identified as adenocarcinomas, diagnosed at stage 1 (36%), and primarily located in the sigmoid colon (27%). The age at diagnosis ranged from 7 to 87 (mean, 60.5) years.

For CRC treatment outcomes, the mean length of hospital stay was 10.3 days, and 77% of patients survived. Comorbidities such as DM and HTN were also recorded. Forty percent of patients had DM, with a mean age of 60.5 years at diagnosis, whereas 53% of the patients had HTN, with a mean age of 61 years at diagnosis.

### 3.2. Comorbidities and patient characteristics

Of the 100 patients, 59 (59%) had at least 1 comorbid illness (DM and/or HTN). Table 2 lists the prevalence of these conditions according to sex, marital status, and smoking status. A higher proportion of males (74.6%) had comorbid conditions compared to females (27.1%). Additionally, a larger proportion of patients with comorbid conditions were married (84.7%) and nonsmokers (54.2%).

**Table 2:** Prevalence of comorbid condition by patient characteristics.

Characteristic		Comorbidity				*P*-value†
		Free		DM and/or HTN		
		Count	%	Count	%	
Total sample		41	41	59	59	–
Sex	Males	22	53.7	44	74.6	**.034**
	Females	19	46.3	15	25.4	
Marital status	Married	28	68.3	50	84.7	.051
	Not married	13	31.7	9	15.3	
Smoking status	Nonsmoker	23	56.1	32	54.2	1.000
	Smoker	18	43.9	27	45.8	

Significance value (*P* < .05) is in bold.

DM = diabetes mellitus, HTN = hypertension.

† Chi-square test.

A chi-square test was performed to assess the relationship between sex, marital status, smoking status, and the presence of comorbid conditions (DM and/or HTN). Sex was significantly associated with comorbidities; male patients more likely to have comorbid conditions than female patients. However, the results revealed no significant association between marital status, smoking status, or the presence of comorbid conditions (*P* > .05). These findings suggested that male patients have a higher risk of comorbid conditions, although marital status and smoking status do not significantly impact this risk.

### 3.3. Comorbidities and colorectal cancer diagnosis and outcomes

The results of the chi-square test between comorbidities (DM and/or HTN), cancer diagnosis, and outcomes (cancer stage, primary site, and patient status) are presented in Table 3.

**Table 3:** Prevalence of comorbid condition by colorectal cancer diagnosis and outcomes.

Characteristic		Comorbidity				*P*-value†
		Free		Had DM and/or HTN		
		Count	%	Count	%	
Total sample		41	41	59	59	–
Cancer stage	Advanced	15	36.6	26	44.1	.537
	Early	26	63.4	33	55.9	
Cancer primary site	Distal colon	34	82.9	44	74.6	.342
	Proximal colon	7	17.1	15	25.4	
Patient status	Alive	36	87.8	42	71.2	**.040**
	Dead	5	12.2	17	28.8	

Significance value (*P* < .05) is in bold.

Cancer stage, Early = stage 1 and 2; Advanced = 3 and 4.

Cancer primary site, Proximal colon = ascending, hepatic flexure, and transverse colon. Distal colon = splenic flexure, descending, sigmoid colon, and rectum.

DM = diabetes mellitus, HTN = hypertension.

† Chi-square test.

Among the 100 patients, 59 (59%) had comorbidities. The proportion of patients with advanced-stage cancer was higher among those with comorbidities (44.1%) compared to those without comorbidities (36.6%). Conversely, the proportion of patients with an early-stage cancer was higher among those without comorbidities (63.4%) than among those with comorbidities (55.9%). However, the presence of comorbidities was not significantly associated with the cancer stage (*P* = .537).

The primary cancer site was also not significantly associated with comorbidities (*P* = .342). However, a significant association was noted between patient status (alive or dead) and comorbidities (*P* = .040). The proportion of surviving patients was higher among those without comorbidities (87.8%) compared to those with comorbidities (71.2%), suggesting that patients with comorbid conditions may have an increased risk of death after a CRC diagnosis.

### 3.4. Comorbidities and colorectal cancer treatment

**Figure 1.**
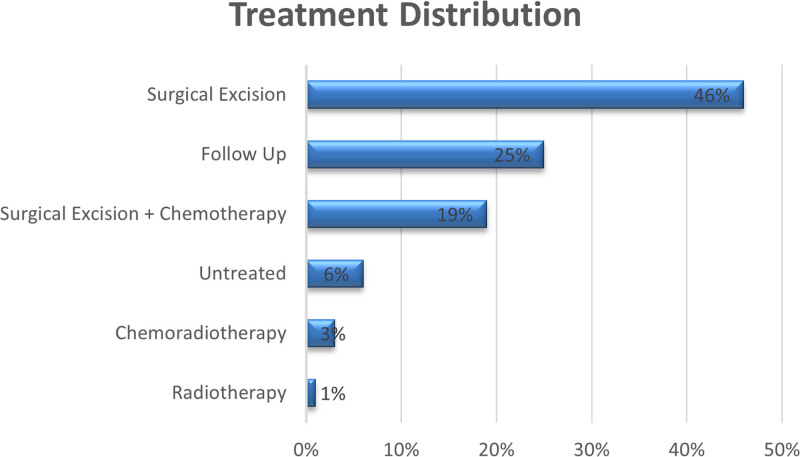
Treatments received by patients with colorectal cancer.

We analyzed various treatment options for colorectal cancer including surgical excision, chemotherapy, radiotherapy, or a combination of these. Figure 1 shows that 46% of patients underwent surgical excision, and 19% underwent surgical excision combined with chemotherapy. Chemoradiotherapy and radiotherapy were administered to 3% and 1% of patients, respectively. Six percent of patients did not receive any treatment and 25% required follow-up at the clinic.

**Figure 2.**
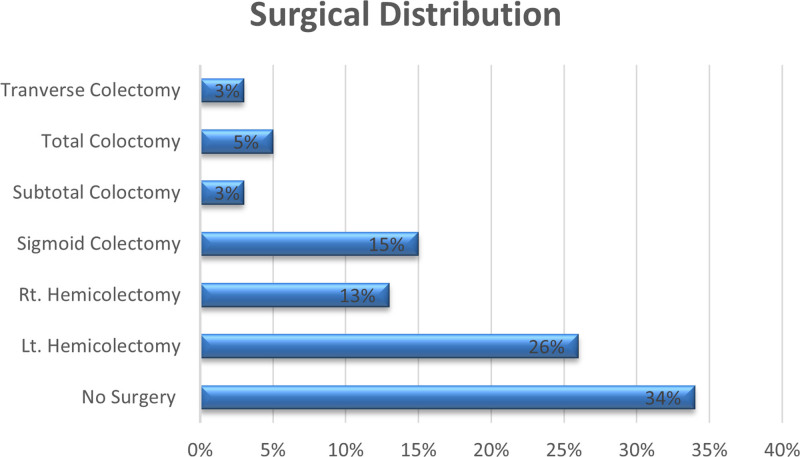
Surgical interventions received by patients with colorectal cancer. Rt., right; Lt., left.

Of the patients who underwent surgical excision, 26 underwent left hemicolectomy, 13 right hemicolectomy, 15 sigmoid colectomy, 3 subtotal colectomy, 5 total colectomy, and 3 transverse colectomies (Fig. 2).

Of the patients with comorbidities, 71.2% underwent surgery, whereas 28.8% did not. In comparison, 56.1% of patients without comorbidities underwent surgery and 34.9% did not. The results of the chi-square test indicated that the presence of comorbidities was not significantly associated with surgery (*P* = .12) (Table 4).

**Table 4:** Prevalence of comorbid condition by surgery.

Receipt of surgery	Comorbidity				*P*-value†
	Free		DM and/or HTN		
	Count	%	Count	%	
No	18	34.9	17	28.8	.139
Yes	23	56.1	42	71.2	

Significant values (*P* < .05).

DM = diabetes mellitus, HTN = hypertension.

† Chi-square test.

## 4. Discussion

In this study, we collected and analyzed data from 100 patients diagnosed and managed for colorectal cancer from Prince Hamza Hospital in Jordan. Our investigation explored the relationship between the number of comorbidities and age in relation to cancer site, as well as to investigated associations with cancer stage, mortality risk, and smoking habits. Previous research on colorectal cancer has shown high rates of comorbid illnesses among patients with cancer, with 88% reporting at least 1 comorbidity. Despite the anticipated increase in the number of people aged 65 years and older and the age-relationship between cancer and other chronic illnesses, there is limited data on whether older cancer patients have a greater prevalence of comorbid conditions compared to older patients without cancer.

In Jordan, CRC is the most common cancer among males, and the second most common cancer among females, accounting for 15% and 9.4% of all male and female diagnosed cancers, respectively. Age is a major risk factor for cancer, with increasing age correlating with higher cancer incidence rates, and a greater prevalence of multiple health issues among older individuals.^[10]^ Comorbidity, defined as a medical condition that exists along with an index condition, is an issue of growing importance owing to demographic shifts and the rising number of adults over the age of 65 with cancer.^[11]^

In one study, over 75% of participants had 2 or more medical conditions, and nearly a quarter of participants had 4 or more conditions, revealing that hypertension, hyperlipidemia, and anemia were the top 3 comorbid illnesses in older adults. Older adults with cancer often have higher rates of comorbidities compared to an age-matched control group without cancer. More than half of all older adults with cancer have at least 1 comorbidity that may impact their cancer treatment, with observational studies revealing that patients with cancer and comorbidities have poorer survival than those without comorbidities. The survival rates of these patients vary by cancer type, and patients with higher rates of comorbidities have a 1.2- to 6-fold higher mortality risk. In a recent cohort study of older adults with cancer, the 5-year all-cause mortality rate rose with an increase in comorbidities across various cancer types, including breast, lung, colorectal, prostate, and ovarian cancers.^[11]^ In another cohort study, the overall sample included patients with and without cancer, mean age of 74 years, predominantly white, married, and possessing a high school education or lower; approximately 47% of cancer patients with cancer were diagnosed more than 5 years previously (mean 6 years, standard deviation 5.6). Patients with cancer in that sample reported a higher prevalence of 10 of the 12 comorbid illnesses, compared to those without cancer.^[12]^

In a separate cohort study of 4 cancers, England cancer registry records of patients diagnosed with cancer of the colon, rectum, lung, or Hodgkin lymphoma between 2009 and 2013 were linked with hospital records; comorbidity was most prevalent in patients with lung cancer and least prevalent in patients with Hodgkin lymphoma. Approximately 75% of patients within each of the 4 cancer cohorts studied had at least 1 comorbid condition, and approximately 50% of these patients had more than 1 comorbidity.^[13]^

The association between cancer and comorbidities has been studied in Jordan, although not extensively. Therefore, our research may provide an approximation of the percentage of comorbidities among patients with colorectal cancer in Jordan and aid in the identification of the most common comorbidities associated with this disease. Studies have shown that the percentage of comorbidities among patients with cancer is much higher than that in non-cancer patients and that there is a direct relationship between the number of comorbidities and aging, indicating that as the population continues to age, the prevalence of comorbidities in oncology grows.

## 5. Conclusion

In this study, the most common anatomical site of colon cancer was the sigmoid colon, and the most common comorbidity among patients with colorectal cancer was hypertension, followed by diabetes mellitus. Mortality was higher among patients with comorbidities. Colorectal cancer is more common in males with a male-to-female ratio (2:1). Therefore, a better understanding of the impact of comorbidities on cancer care and management strategies is essential. Moreover, these conditions require special precautions to avoid potential negative interactions and unexpected adverse reactions to prescribed cancer-specific treatments, and underscores the need for multidisciplinary care and treatment decisions.

## Acknowledgments

We would like to thank Editage (www.editage.com) for English language editing.

## Author contributions

**Conceptualization:** Jehad Fataftah, Naser El-Hammuri, Abdel Rahman Alfawadleh, Ayman Alqubelat.

**Data curation:** Jehad Fataftah, Mohammad Badran, Aqleh Ibrahim, Basil Alramahi, Abdel Rahman Alfawadleh, Ayman Alqubelat.

**Formal analysis:** Jehad Fataftah, Naser El-Hammuri, Mohammad Badran, Aqleh Ibrahim, Basil Alramahi, Abdel Rahman Alfawadleh, Ayman Alqubelat.

**Investigation:** Jehad Fataftah, Naser El-Hammuri, Ayman Alqubelat.

**Methodology:** Jehad Fataftah, Naser El-Hammuri, Mohammad Badran.

**Project administration:** Jehad Fataftah.

**Resources:** Jehad Fataftah.

**Software:** Mohammad Badran, Basil Alramahi.

**Supervision:** Jehad Fataftah, Maha Gharaibeh, Aqleh Ibrahim, Abdel Rahman Alfawadleh, Ayman Alqubelat.

**Validation:** Jehad Fataftah, Naser El-Hammuri, Mohammad Badran, Basil Alramahi, Abdel Rahman Alfawadleh, Ayman Alqubelat.

**Visualization:** Jehad Fataftah.

**Writing – original draft:** Jehad Fataftah, Mohammad Badran, Aqleh Ibrahim.

**Writing – review & editing:** Jehad Fataftah, Naser El-Hammuri, Maha Gharaibeh.
